# Assessment of diagnostic accuracy of reveal^®^ autofluorescent dental loupes for detection of biofilm, demineralization, and caries around orthodontic brackets

**DOI:** 10.1590/2177-6709.30.4.e252577.oar

**Published:** 2025-11-07

**Authors:** Filipe do Carmo BONFANTE, Anna Vithoria da Costa LONGHI, Marianna DEMARCHI, Dora Marise Medeiros de CASTRO, Fernando Kleinubing RHODEN, Gabriel Barcelos SÓ, João Paulo de CARLI, Pedro Henrique CORAZZA, Yuri Dal BELLO, Liviu STEIER, José Antonio Poli de FIGUEIREDO, Matheus Albino SOUZA

**Affiliations:** 1 Universidade de Passo Fundo, Faculdade de Odontologia (Passo Fundo/RS, Brazil).; 2 Instituto Rhoden, Faculdade de Odontologia (Passo Fundo/RS, Brazil).; 3 Universidade Federal do Rio Grande do Sul, Faculdade de Odontologia (Porto Alegre/RS, Brazil).; 4 University of Pennsylvania, Dental Medicine School (Philadelphia, USA).; 5 Saveetha Institute of Medical and Technical Sciences, Saveetha Dental College and Hospitals (Chennai, India).

**Keywords:** Biofilm, Caries, Demineralization, Diagnosis, Fluorescence, Orthodontics, Biofilme, Cárie, Desmineralização, Diagnóstico, Fluorescência, Ortodontia

## Abstract

**Introduction:**

The aim of the present study was to evaluate the diagnostic accuracy of Reveal autofluorescent dental loupes for detecting biofilm, demineralization, and caries around orthodontic brackets.

**Methods:**

Sixty patients were selected, being thirty patients without orthodontic treatment (control group) and thirty patients undergoing orthodontic treatment (experimental group). The 30 patients without orthodontics treatment were clinically evaluated by two previously calibrated evaluators, who assessed the presence of biofilm, demineralization, and incipient caries on the buccal surface of the upper anterior teeth by using two methods: G1 (n=30)-visual method; G2 (n=30)-Reveal^®^ method. The 30 patients with orthodontic treatment were evaluated in the same way. In both groups, a scoring system was assigned to evaluate the variables of the study. For statistical analysis, an average score for each patient was calculated by adding the scores for each tooth and dividing by the number of teeth evaluated. A statistical comparison was performed by the Wilcoxon signed-ranks test (α = 0.05).

**Results:**

The Reveal method was more effective at detecting biofilm in patients without orthodontic treatment (p < 0.05). There were no statistically significant differences between the Reveal method and the visual method in detecting demineralization and caries (p > 0.05). Furthermore, the Reveal method was more efficient in detecting biofilm and demineralization in patients with orthodontic treatment (p < 0.05) and showed no statistically significant differences compared to the visual method in detecting caries (p > 0.05).

**Conclusions:**

The Reveal autofluorescent dental loupes effectively detected biofilm, demineralization, and incipient caries around orthodontic brackets.

## RESUMO

**Introdução:**

O objetivo do presente estudo foi avaliar a precisão do diagnóstico das lupas dentárias autofluorescentes Reveal para detectar biofilme, desmineralização e cárie ao redor de braquetes ortodônticos.

**Métodos:**

Foram selecionados 60 pacientes, sendo 30 pacientes sem tratamento ortodôntico (grupo controle) e 30 pacientes submetidos a tratamento ortodôntico (grupo experimental). Os 30 pacientes sem tratamento ortodôntico foram avaliados clinicamente por dois avaliadores previamente calibrados, que avaliaram a presença de biofilme, desmineralização e cárie incipiente na superfície vestibular dos dentes anteriores superiores por meio de dois métodos: G1 (n=30) - método visual; G2 (n=30) - método Reveal®. Os 30 pacientes com tratamento ortodôntico foram avaliados da mesma forma. Em ambos os grupos, foi atribuído um sistema de pontuação para avaliar as variáveis do estudo. Para a análise estatística, foi calculado um valor de escore médio para cada paciente, somando os escores de cada dente e dividindo pelo número de dentes avaliados. A comparação estatística foi realizada pelo teste Wilcoxon Signed Ranks (α=0,05).

**Resultados:**

O método Reveal foi mais eficaz na detecção da presença de biofilme nos pacientes sem tratamento ortodôntico (p<0,05), sem diferenças estatisticamente significativas em relação ao método visual na detecção de desmineralização e cárie (p>0,05). Além disso, o método Reveal foi mais eficiente na detecção da presença de biofilme e desmineralização nos pacientes com tratamento ortodôntico sem diferenças estatisticamente significativas em relação ao método visual na detecção de cáries (p>0,05).

**Conclusões:**

As lupas dentárias autofluorescentes Reveal foram eficazes na detecção de biofilme, desmineralização e cáries incipientes ao redor de braquetes ortodônticos.

## INTRODUCTION

Orthodontic treatment involves moving teeth and jaw bones with brackets and wires to assist in repositioning them. The main objectives are to restore occlusal balance, mastication function, and aesthetics for the patient.^1^ The average duration of orthodontic treatment ranges from 1 to 3 years, depending on the case. The bonding of the orthodontic appliance represents a risk factor for poor oral hygiene due to the high accumulation of plaque around the brackets and orthodontic wires and the difficulty in cleaning these areas.^2^ Consequently, the demineralization of tooth enamel and the progression to dental caries can occur,^3^ as well as periodontal damage due to the significant presence of microbial biofilm.^2^ Therefore, early diagnosis of these pathological changes in dental tissues is essential for maintaining oral health.

The visual method is the most commonly used resource for evaluating the oral hygiene standards of patients undergoing orthodontic treatment. This is done through direct vision of the human eye, with the help of a front-surface mirror and the conventional light source of the reflector, whether halogen or LED.^4^ However, this method can be considered subjective, since the level of perception among professionals varies, and the initial stages of microbial biofilm accumulation, enamel demineralization, and incipient caries around orthodontic brackets are difficult to detect.^3^ It is also subject to error, because these early subsurface lesions present no cavitation and the enamel surface remains intact, making it difficult to diagnose during their initial stages.^3^ In addition, remineralization measures can only be used effectively if demineralization is initially detected.^5^ Finally, the orthodontic appliance and its accessories obstruct and make it difficult for the human eye to assess any changes in the dental tissues.^4^ In this scenario of several limitations of visual method, it is necessary to use more accurate auxiliary diagnostic resources to detect and prevent the progression of these pathological lesions in orthodontic patients early on.

Fluorescence technology represents an innovative diagnostic resource that detects pathological lesions in tooth enamel.^6^ This technology involves the absorption and re-emission of light at different wavelengths, offering significant insights into mineralization variations in dental tissues.^7^ Furthermore, fluorescence technology refers to the intrinsic fluorescence emitted by biological tissues or microorganisms when exposed to light of a specific wavelength.^8^ Recently developed, the Reveal^®^ autofluorescent dental loupes (Designs for Vision, Bohemia, NY, USA) combine magnification with the emission of fluorescent light. They have emerged as a valuable auxiliary method for detecting dental caries,^9^ cracked teeth,^10^ dental biofilm,^11^ oral potentially malignant disorders, and oral cancer.^12^ However, few studies reveal this device’s effectiveness in detecting biofilm, demineralization, and caries around brackets in patients undergoing orthodontic treatment. Considering the innovative and technological characteristics of Reveal^®^ device, it can be evaluated, in order to replace the visual method, as well as provide greater magnification, clarity and diagnostic accuracy, helping to maintain oral health and contributing to the adequate treatment of the orthodontic patients.

This study aimed to evaluate the diagnostic accuracy of the Reveal^®️^ autofluorescent dental loupes for detecting biofilm, demineralization, and incipient caries around orthodontic brackets. The null hypotheses of this study were that Reveal^®️^ device (i) is less effective in detecting biofilm, (ii) demineralization, and (iii) incipient caries around orthodontic brackets when compared to the visual method.

## MATERIAL AND METHODS

### SAMPLE SIZE

This study was approved by the local Research Ethics Committee (protocol 6.515.846) and registered at local Clinical Trial Registry (ReBEC) for clinical studies. The BioEstat 5.0 statistical package (Fundação Mamirauá, Belém, PA, Brazil) was used to calculate the sample size, requiring a minimum of 12 patients per group to be included in the present study, considering a test power of 80% and alpha error of 5%, in order to estimate the sample size for analysed outcomes. Although the sample size calculation indicated the need for 12 patients per group for the present study, 60 patients were selected, being 30 patients with no orthodontic treatment (control group) and 30 patients with orthodontic treatment (experimental group). It ensures greater potential reliability of the results of the present study.

Among the inclusion criteria for both groups, patients aged ≥ 18 years with good general health were included in the present study. Furthermore, for the experimental group, patients undergoing orthodontic treatment for at least one year from the bracket bonding appointment and presenting orthodontic brackets on the upper anterior teeth were included in the present study. All potential participants were informed verbally and in writing, and they signed an informed consent form. Among the exclusion criteria for both groups, patients who had significant disabilities that affected manual dexterity or oral hygiene practice, who had been taking antibiotics in the last two months, or who refused to sign the informed consent form were excluded from the study.

### GROUP DISTRIBUTION AND EVALUATION METHOD

**Figure 1:**
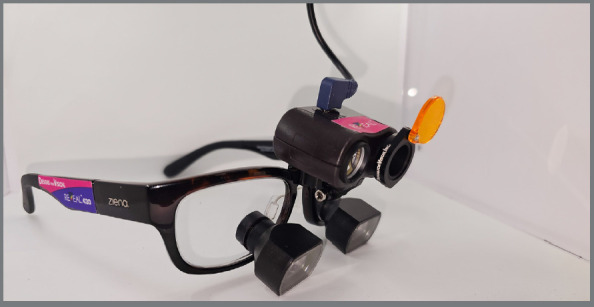
Illustration of Reveal®️ autofluorescent dental loupes.

The 30 patients of the control group (with no orthodontic treatment) were clinically evaluated by two previously calibrated evaluators, who assessed the presence of biofilm, demineralization, and incipient caries on the buccal surface of upper anterior teeth using two methods:

» Group 1 (n = 30-visual method: The visual field was dried using relative isolation, an air jet, and white LED light from the dental chair reflector.
» Group 2 (n = 30)-Reveal^®️^ method: by using relative isolation, visual field dried with jet air and autofluorescence induced by the Reveal^®️^ device (Designs for Vision, Bohemia, NY, United States). Figure 1 illustrates the Reveal^®️^ device.

Regarding the assessment of the presence of biofilm in both methods, a numerical score was assigned to each one of the six upper anterior teeth evaluated, obtaining six numerical scores per patient of each group, as follows:

Score 0: no presence of plaque (visual) or orange fluorescent area (Reveal^®️^) on the buccal surface of the dental element; Score 1: presence of plaque (visual) or orange fluorescent area (Reveal^®️^) on the buccal surface of the tooth.

Regarding the assessment of the presence of demineralization in both methods, dental prophylaxis was carried out in order to remove the microbial biofilm. After this, a numerical score was assigned to each one of the six upper anterior teeth evaluated, obtaining six numerical scores per patient of each group, as follows:

Score 0: There is no thick white spot (visual) or brown fluorescent area (Reveal^®️^) on the buccal surface of the tooth; Score 1: There is a thick white spot (visual) or brown fluorescent area (Reveal^®️^) on the buccal surface of the tooth.

Regarding the assessment of the presence of incipient caries in both methods, a numerical score was assigned to each one of the six upper anterior teeth evaluated, obtaining six numerical scores per patient of each group, as follows:

Score 0: There is no decayed tissue (visual) or red/orange fluorescent area (Reveal^®️^) on the buccal surface of the tooth; Score 1: There is a decayed tissue (visual) or red/orange fluorescent area (Reveal^®️^) on the buccal surface of the tooth.

The remaining 30 patients of the experimental group (with orthodontic treatment) were clinically evaluated by the same two previously calibrated evaluators, who assessed the presence of biofilm, demineralization, and incipient caries on the buccal surface of upper anterior teeth using the same two methods, as previously described, being G1-visual method; and G2-Reveal^®️^ method. 

Regarding the assessment of the presence of biofilm in both methods, a numerical score was assigned to each one of the six upper anterior teeth evaluated, obtaining six numerical scores per patient of each group, as follows:

Score 0: no presence of plaque (visual) or orange fluorescent area (Reveal^®️^) around the orthodontic bracket on the buccal surface; Score 1: presence of plaque (visual) or orange fluorescent area (Reveal^®️^) on one of the four sides of the orthodontic bracket (25%); Score 2: presence of plaque (visual) or orange fluorescent area (Reveal^®️^) on two of the four sides of the orthodontic bracket (50%); Score 3: presence of plaque (visual) or orange fluorescent area (Reveal^®️^) on three of the four sides of the orthodontic bracket (75%); Score 4: presence of plaque (visual) or orange fluorescent area (Reveal^®️^) on all four sides of the orthodontic bracket (100%). 

Regarding assessing the presence of demineralization in both methods, dental prophylaxis was carried out to remove the microbial biofilm. After this, a numerical score was assigned to each one of the six upper anterior teeth evaluated, obtaining six numerical scores per patient of each group, as follows:

Score 0: no presence of thick white spot (visual) or brown fluorescent area (Reveal^®️^) around the orthodontic bracket on the buccal surface; Score 1: the presence of thick white spot (visual) or brown fluorescent area (Reveal^®️^) on one of the four sides of the orthodontic bracket (25%); Score 2: the presence of thick white spot (visual) or brown fluorescent area (Reveal^®️^) on two of the four sides of the orthodontic bracket (50%); Score 3: presence of thick white spot (visual) or brown fluorescent area (Reveal^®️^) on three of the four sides of the orthodontic bracket (75%); Score 4: presence of thick white spot (visual) or brown fluorescent area (Reveal^®^) on all four sides of the orthodontic bracket (100%).

Regarding the assessment of the presence of incipient caries in both methods, a numerical score was assigned to each one of the six upper anterior teeth evaluated, obtaining six numerical scores per patient of each group, as follows:

Score 0: no presence of decayed tissue (visual) or red/orange fluorescent area (Reveal^®️^) around the orthodontic bracket on the buccal surface; Score 1: presence of decayed tissue (visual) or red/orange fluorescent area (Reveal^®️^) on one of the four sides of the orthodontic bracket (25%); Score 2: presence of decayed tissue (visual) or red/orange fluorescent area (Reveal^®️^) on two of the four sides of the orthodontic bracket (50%); Score 3: presence of decayed tissue (visual) or red/orange fluorescent area (Reveal^®️^) on three of the four sides of the orthodontic bracket (75%); Score 4: presence of decayed tissue (visual) or red/orange fluorescent area (Reveal^®️^) on all four sides of the orthodontic bracket (100%).

### STATISTICAL ANALYSIS

For statistical analysis, an average score value was calculated for each patient, adding the scores for each tooth (0 to 4) and dividing by the number of teeth evaluated in the assessments of the presence of biofilm, demineralization, and incipient caries, both in the group of patients without orthodontic treatment and in the group of patients with orthodontic treatment. Two expert evaluators graduated from the Orthodontics residency program were responsible for assigning the scores. The Kappa coefficient test was performed to confirm the inter-rater reliability of the assessment between the both evaluators (k=0.9376).

Statistical comparison was performed by the Wilcoxon Signed Ranks Test (α = 0.05), comparing the visual and Reveal^®️^ device methods based on the establishment of a mean and standard deviation of the numerical score for the methods. The variables biofilm, demineralization and incipient caries were evaluated separately. The Stat Plus AnalystSoft Inc. version 6.0 software (Vancouver, BC, Canada) was used to perform the statistical analysis.

## RESULTS

**Figure 2:**
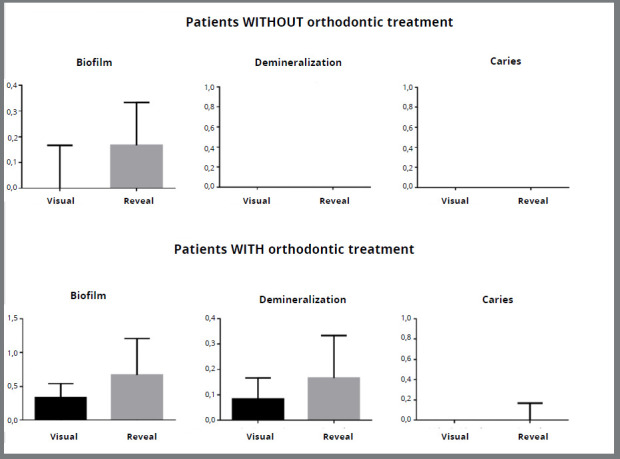
Graph revealing mean and standard deviation of the numerical scores for the visual and Reveal^®^ methods in evaluating the presence of biofilm, demineralization and caries, in patients without and with orthodontic treatment.

**Figure 3:**
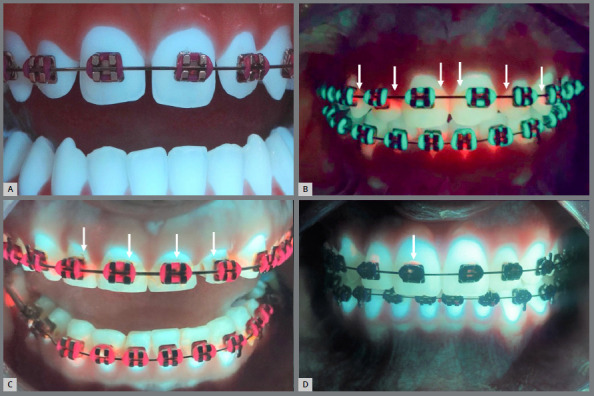
Images obtained by Reveal^®^ autofluorescent dental loupes revealing **(**A) no pathological alteration on dental surface; **(**B) presence of biofilm; **(**C) presence of demineralization; **(**D) presence of incipient caries around orthodontic brackets. The arrows indicate the presence of the observed pathological changes.

The mean and standard deviation of the numerical scores for the visual and Reveal^®^ methods in evaluating the presence of biofilm, demineralization, and caries in patients without and with orthodontic treatment, are expressed in Table 1 and Table 2, respectively. Figure 2 also demonstrates these results. The Reveal^®^ method was more efficient in detecting the presence of biofilm in the patients without orthodontic treatment (p<0.001), with no statistically significant differences to the visual method in the detection of demineralization (p>0.05). The number of patients without orthodontic treatment and with incipient caries did not allow comparison between the methods for this characteristic. The Reveal^®^ method was more efficient in detecting the presence of biofilm and demineralization in the patients with orthodontic treatment (p<0.001 and p=0.004 respectively), with no statistically significant differences from the visual method in the detection of incipient caries (p=0.06). Figure 3 provides an illustration of images obtained by Reveal^®️^ autofluorescent dental loupes, revealing no pathological alteration on the dental surface, the presence of biofilm, the presence of demineralization, and the presence of incipient caries around orthodontic brackets.

**Table 1:** Mean ± standard deviation of the numerical scores for the visual and ReVeal^®^ methods in evaluating the presence of biofilm, demineralization and incipient caries, in patients without orthodontic treatment.

Assessment	n	Visual Method	ReVeal^®^ Method
Biofilm	30	0.07 ± 0.01^B^	0.19 ± 0.02^A^
Demineralization	30	0.01 ± 0.05^A^	0.01 ± 0.05^A^
Incipient caries	30	0.00 ± 0.00^A^	0.00 ± 0.00^A^

* Different uppercase letters, in the row, represents statistically significant differences (p<0.05).

**Table 2:** Mean ± standard deviation of the numerical scores for the visual and ReVeal^®^ methods in evaluating the presence of biofilm, demineralization and incipient caries, in patients with orthodontic treatment.

Assessment	n	Visual Method	ReVeal^®^ Method
Biofilm	30	0.41 ± 0.04^B^	0.90 ± 0.28^A^
Demineralization	30	0.14 ± 0.02^B^	0.23 ± 0.02^A^
Incipient caries	30	0.03 ± 0.08^A^	0.07 ± 0.14^A^

* Different uppercase letters, in the row, represents statistically significant differences (p<0.05).

## DISCUSSION

Orthodontic patients face additional challenges in maintaining oral hygiene due to the presence of these accessories, requiring constant efforts to achieve acceptable levels of oral cleanliness. Likewise, it is the responsibility of the dental clinician to diagnose the significant accumulation of bacterial plaque early, helping with oral hygiene and preventing the evolution of pathological changes in dental tissues. Patient cooperation during orthodontic treatment is essential to maintain satisfactory hygiene of tooth surfaces around orthodontic brackets. Considering this challenge, several approaches have been suggested, including preventive measures, communication methods, and guidance on frequency and hygiene techniques.^13^ Otherwise, there is a greater propensity for the accumulation of bacterial plaque on tooth surfaces close to orthodontic accessories.^14^ In these cases, it is essential to identify areas with more susceptibility to biofilm accumulation, defining preventive and therapeutic strategies that avoid the evolution of pathological changes on these surfaces. In this proposal, visual resources are the most used method for diagnosis. However, it presents limited sensitivity, subjectivity in interpretation, visual limitation in areas that are difficult to access, and the human eye’s limit provides low magnification of the operative field.^15^ For these reasons, the present study proposed the use of a new alternative in the scope of diagnosis in order to detect pathological changes around orthodontic brackets.

Recently, the Reveal^®^ device (Designs for Vision, Bohemia, NY, USA) was developed, offering benefits to the diagnosis and treatment of pathological changes in the oral cavity.^7^ It presents itself as a pair of glasses containing a magnifying glass and a fluorescent light-emitting source attached to the glasses, increasing the visualization of the operative field by 2.5 times and emitting a fluorescent light directed towards the operative field.^16^ When the light is activated, the device can induce photoluminescence due to the intrinsic fluorescence characteristics of the tooth structure that reflect demineralization.

Furthermore, it induces the bioluminescence of bacterial components and by-products, making it possible to diagnose microorganisms when they are in total activity.^7^ ^,^ ^17^ ^,^ ^18^ In this way, relevant visual information is provided to support clinical decisions in several areas of dentistry, from diagnosis to treatment completion. Unlike other fluorescent light-emitting resources, the Reveal^®^ presents easy portability, hands-free design, and the benefit of high-precision magnification, offering a practical solution for clinical applications. For these reasons, the present study used the chosen method to evaluate the presence of biofilm, demineralization, and incipient caries around orthodontic brackets.

According to the results of the present study, the Reveal^®️^ device was more efficient in detecting the presence of microbial biofilm, both in the group of patients without orthodontic treatment and in the group of patients undergoing orthodontic treatment. These findings reject the first null hypothesis of the present study. In addition to the magnification of the operative field, which enhances the visualization ability of the microbial biofilm, bacteria irradiated with the device’s fluorescent light emit photons in the orange wavelength range due to the presence of porphyrin derivatives in their structural composition, which is a class of organic compounds present in all living bacterial cells.^19^

Additionally, it was observed that the more mature biofilm emits an orange color when it is active,^15^ ^,^ ^18^ ^,^ ^20^ corroborating the present study’s findings. Due to the fluorescent properties of the porphyrins, active microbial biofilm is easily identified when present by visualizing orange fluorescent areas around orthodontic brackets when using the Reveal^®^ device. This does not occur when we use the visual method, which, in addition to providing a restricted view of the operative field due to the limitations of the human eye, does not present the ability to visualize these forms of irradiation that facilitate the identification of the microbial biofilm. It helps explain the results of the present study.

When the Reveal^®^ device is used, enamel demineralization appears in the form of a brown color. This occurs due to the increased dispersion of the fluorescent light emitted by the Reveal^®^ device over the affected tissue, resulting in the visualization of brown coloration in these demineralized areas.^9^ ^,^ ^21^ ^,^ ^22^ According to the results of the present study, the Reveal^®^ device was more efficient in detecting the presence of enamel demineralization in the group of patients undergoing orthodontic treatment. These findings reject the second null hypothesis of the present study. In addition to the reasons previously mentioned that explain greater diagnostic accuracy of changes in dental tissues using the Reveal^®^ device, the fact of the diagnostic difficulty of the visual method is added. The presence of biofilm and white spots, indicating the initial stages of enamel demineralization, are confused in the visual method, making the diagnosis even more limited. Therefore, by simply visualizing brown fluorescent areas, the diagnosis of enamel demineralization is more effective using the Reveal^®^ device, as observed in the present study. On the other hand, no significant differences were observed between the visual method and the Reveal^®^ method when it came to detecting demineralization in patients without orthodontic treatment. It can be explained by the fact that anterior teeth are easier to clean when compared to posterior teeth, especially when these patients do not have additional biofilm retainers, such as brackets and orthodontic accessories.

According to previous studies, directing a fluorescent light source at a specific wavelength over decayed tissue with active microorganisms induces a reflection of a red/orange color. The reflection of this color is also due to the presence of porphyrins in the structural components of bacteria responsible for the development of caries disease.^9^ ^,^ ^18^ ^,^ ^20^ ^-^ ^22^

Given the above, using the Reveal^®^ device in the present study could bring greater accuracy in detecting incipient caries in both patient groups, whether they are undergoing orthodontic treatment or not. However, despite the red/orange irradiations observed in some of the evaluated patients, no significant differences were found in the visual method used for this purpose in either group. These findings confirm the third null hypothesis of the present study. It can also be explained by the previously explained reasons, where greater anterior teeth cleaning brings a lower incidence of more aggressive pathological changes in these regions, as observed in the incipient caries evaluation. It is possible that the Reveal^®^ device would bring more precision in the detection and delimitation of incipient caries in posterior teeth, as well as in more advanced stages of this pathological alteration on dental enamel.

The existence of novelty and more accurate method for early detection of the pathological changes in the dental tissues brings several benefits to orthodontic patients. One of the main advantages is the possibility of performing less invasive interventions. If biofilm, demineralized tissue, or cavities are identified in the early stages, the process can be reversed or stabilized with preventive measures, such as prophylaxis, application of fluoride and oral hygiene instructions. As a result, early diagnosis can prevent cavities from progressing to more serious conditions and eliminate the need for extensive, invasive restorations.

In addition, the therapeutic procedure can also be carried out with magnification and fluorescence guidance, without the use of hands and without inducing tissue aggression. It provides bringing high precision in the removal of the bacterial biofilms, demineralized tissue and carious lesions is more effective.^16^ ^-^ ^18^ Thus, it is possible to conservatively remove the infected tissue and provide excellent preservation of the healthy dental structure by using the Reveal^®^ autofluorescent dental loupes. This set of factors contributes to the establishment of a strategy to ensure health, efficiency and quality of life during orthodontic treatment.

Regarding the limitations of the present study, it was observed that patients tend to clean their front teeth more frequently and effectively due to their visibility and accessibility during brushing. This may have influenced the results of the present study, especially when assessing the presence of incipient caries. Additionally, patients with red/orange bandages around their orthodontic brackets made photographic record-taking difficult. Evaluating the posterior teeth could provide more significant differences when comparing the two methods.

This is the first study to use the Reveal^®️^ device and loupes on orthodontic appliances. It allows advances in the diagnosis of the presence of biofilm in patients undergoing orthodontic treatment. High levels of orange fluorescence around orthodontic brackets when using the Reveal^®^ device can be an effective indicator of the progression of bacterial lesions in dental tissues. Moreover, this autofluorescent technology can be explored into the several fields of dentistry, expanding its application in further studies.

The severity and degree of unmet dental needs are significant among the population^23^ and oral hygiene protocols are necessary to decrease these levels. The incorporation of nanoparticles into orthodontic appliances and restorative materials holds promise for enhancing their antibacterial properties.^24^ ^,^ ^25^ Different areas of dentistry that are directly related to the presence of microorganisms, such as cariology, periodontics, endodontics and implantology, as well as stomatology that lacks more precise methods for detecting tumor tissue. All these practice scenarios can be contemplated with the use of the Reveal^®️^ device, both in diagnostic and in therapeutic evaluations of different protocols. On this way, more reliable results, innovation and technology will provide significant contribution to high-quality dentistry at all levels.

## CONCLUSIONS

Despite the limitations of the present study, the Reveal^®️^ autofluorescent dental loupes were effective for detecting biofilm, demineralization, and incipient caries around orthodontic brackets.
